# Congenital pulmonary airway malformation complicated by aspergilloma: A rare adulthood presentation - Case report

**DOI:** 10.1016/j.mmcr.2023.07.001

**Published:** 2023-07-22

**Authors:** Rihab Molah, Nasser Altowairqi, Bader Alotaibi, Ali Alzughbi, Hanaa Bamefleh

**Affiliations:** a College of Medicine, King Saud University, Riyadh, 13315, Saudi Arabia; b King Saud Bin Abdualziz University for Health Sciences, Riyadh, 11481, Saudi Arabia; c King Fahad Medical City, Riyadh, 11525, Saudi Arabia

**Keywords:** Aspergilloma, Congenital pulmonary airway malformation, Chronic cavitary pulmonary aspergillosis, CCAM, CPAM

## Abstract

Congenital Pulmonary Airway Malformation (CPAM) is an uncommon condition in adults, which typically presents as acute fever and lung abscesses caused by bacterial infections. We present a case of a 39-year-old female with a CPAM in the upper lobe of the right lung, complicated by an aspergilloma, who presented with a history of hemoptysis. The patient underwent an upper lobectomy and is symptom-free in follow-up. 2012 Elsevier Ltd. All rights reserved.

## 1 Introduction

Congenital Pulmonary Airway Malformation, formerly known as Congenital Cystic Adenomatoid Malformation (CCAM), is a congenital disease in which adenomatic hyperplasia of the bronchiolar epithelium leads to the formation of multiple cysts. In 1977, Stoker et al. [1] classified CCAM lesions into types I–III, and in 1994, he proposed the term congenital pulmonary airway malformation “CPAM” and defined it as a hamartomatous disease that can arise in any part of the tracheobronchial tree. He further expanded its classification to encompass the 5 types: 0–IV. Approximately 80% of CPAMs are detected and diagnosed prenatally or during the neonatal period when patients initially present with respiratory failure and cyanosis. Most CPAMs that are not identified during the neonatal period are detected in infants and school-age children, and these diagnoses are often triggered by infections, such as pneumonia. CPAMs are rarely discovered in adults and usually involve a unilateral lobe of the lung, triggering the same manifestations as in neonates and children [[2], [3], [4]]. Additionally, CPAM complicated by aspergilloma is rather rare, having only been reported in a handful of cases [2,[5], [6], [7], [8], [9]]. Here, we describe a rare case of an adult female with CPAM type 1, which was complicated with pulmonary aspergilloma.

## 2 Case presentation

The patient initially presented during her adolescence with symptoms consistent with respiratory allergies and one episode of hemoptysis at a different hospital. Despite initial conservative antibiotic therapy and diagnostic tests, such as tuberculosis and a plain chest X-ray (CRX), her symptoms persisted. She was eventually diagnosed with an asthmatic condition and treated symptomatically for several years.

At the age of 39, she visited a hospital with access to computerized tomography (CT) scans, she was diagnosed with pulmonary aspergilloma and advised to consult with a thoracic surgeon. She later presented at our tertiary care hospital with a chest CT scan confirming the diagnosis of pulmonary aspergilloma.

**Fig. 1:**
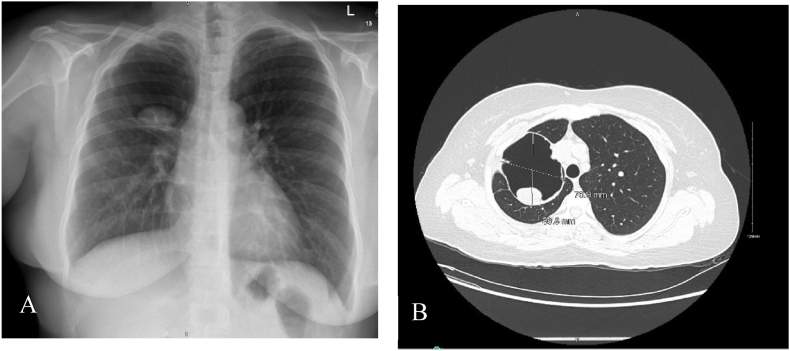
(A) Chest X-Ray with large cyst in the right upper lobe. (B) CT Scan shows the cyst with persistent internal solid component and new adjacent air fluid level and internal air foci.

At our center, we repeated imaging studies. Her chest CXR showed a cystic lesion in the right upper lobe of her lung (Fig. 1-A). Comparison of this CT scan with her prior scan from another hospital revealed a persistence of the cystic lesion with an internal solid component, air-fluid levels, and internal air foci (Fig. 1-B). The morphological appearance was consistent with a cavitary lesion containing an aspergilloma, which was classified as day 0 of her medical history. On day 3, the patient was prescribed oral antifungal therapy (Itraconazole) at a dosage of 200 mg per day, to be taken for 60 days.

A CT scan performed on day 112 revealed the same findings, despite the previous antifungal therapy. The same antifungal therapy course was repeated on days 108 and 166, but the patient's symptoms persisted. No sputum culture was ever done. Surgical treatment was eventually decided on day 167 and the patient underwent a right video-assisted thoracoscopic surgery, which resulted in postoperative pneumothorax and subcutaneous emphysema. Two chest tubes were inserted and remained for 17 days. On day 184 post-surgery, both the emphysema and pneumothorax had improved, and the chest tubes were removed.

**Fig. 2:**
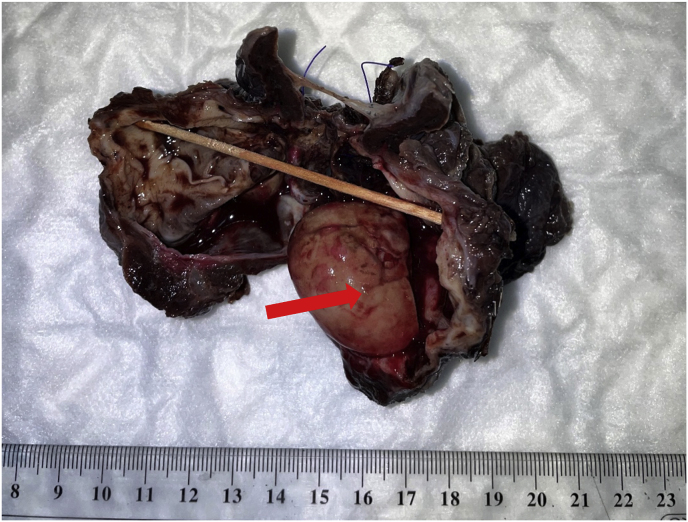
Gross appearance of the right lung lobe with the cyst opened containing tan, ball-like structure (Arrow).

**Fig. 3:**
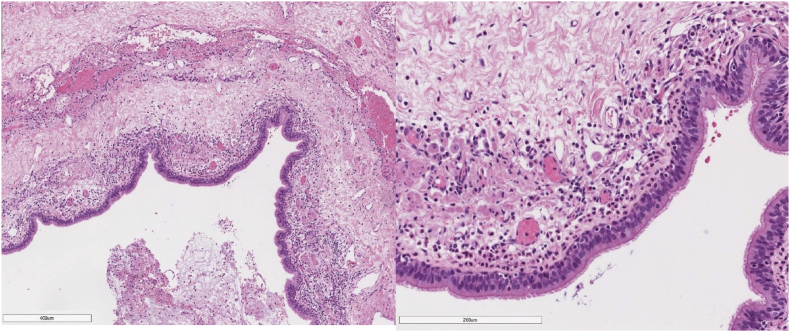
The cyst is lined by pseudostratified ciliated columnar epithelium as shown on low power (right) and intermediate power (left).

**Fig. 4:**
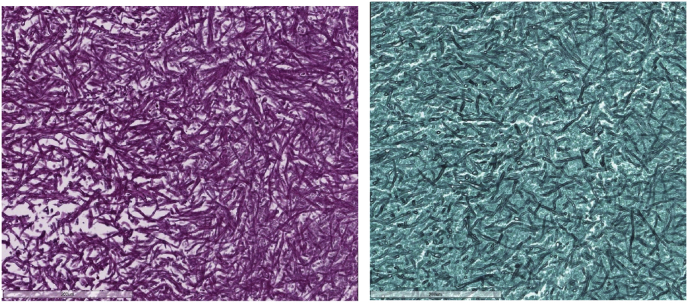
The fungal hyphae are stained by Periodic acid–Schiff (PAS), (left) And Grocott Methenamine Silver (GMS), (right) stains.

The excised specimen for the patient's right upper lobe was measured to weigh 76.2 g and measured 10.0 cm × 8.0 cm x 3.5 cm. Upon cross section, a cyst measuring 7.0 cm × 4.5 cm x 3.5 cm was observed, with a trabeculated wall of tan color. Within the cyst, a laminated, ball-like structure with a tan coloration was discovered (Fig. 2). A microscopic examination revealed that the cyst was lined with pseudostratified ciliated columnar epithelium and contained a ball of fungi with acute angles, dichotomous branching, and septate hyphae. The fungi appeared positive on GMS and PAS staining (see Fig. 3, Fig. 4). On day 297, a galactomannan test for aspergillosis was performed on the patient's sputum, but no detection was made. On day 299, a culture for AFB produced a negative result.

Post-surgery, the patient was placed on a respiratory physiotherapy program. On her last visit to the clinic, which occurred on day 502, the patient was reported to be doing well with no respiratory symptoms. An examination of her chest x-ray showed full expansion of her right lung.

## 3 Discussion

Chronic pulmonary aspergillosis (CPA) is a slowly progressive, destructive lung disease caused by a fungal infection of the lung, caused by members of the Aspergillus genus. It is characterized by pulmonary and/or systemic symptoms, cavitation of the lungs, and the presence of positive Aspergillus-specific IgG antibodies in the serum [[10], [11], [12]]. The most common symptom is hemoptysis, which is reported to be seen in 50–80% of patients. Most patients will experience mild hemoptysis, but massive and life-threatening hemoptysis may occur, particularly in patients with underlying post-tuberculosis [13]. Chronic pulmonary aspergillosis encompasses a number of different disease presentations, with considerable overlap between them, making diagnosis challenging. Of these different types of CPA, Chronic Cavitary Pulmonary Aspergillosis (CCPA), formerly known as Complex Aspergilloma, is the most prevalent. Typically, it manifests as multiple cavities, some of which may contain aspergillomas, as well as pulmonary, systemic, and elevated inflammatory markers. If left untreated, it can progress to chronic fibrosing pulmonary aspergillosis. In addition to Aspergillus nodules and solitary Aspergilloma - two less common types of CPA - these three entities were found to be present in non-immunocompromised patients with prior or ongoing respiratory illnesses [14]. Aspergilloma is a fungal ball composed mainly of extracellular matrix and fungal hyphae, located within an intrathoracic cavity, usually in the parenchyma of the lung, but occasionally found in the pleura or bronchus. A computed tomography (CT) scan of the thorax typically reveals CPA in a pulmonary cavity, though it can rarely be seen in the pleural cavity or an ectatic bronchus [11,14]. This imaging feature is the most distinctive of CPA. Aspergilloma is highly characteristic of CPA and can be found in all forms except Aspergillus nodule. Also, it can develop in a pre-existing cavity, as well as in a new cavity formed during the course of untreated chronic pulmonary aspergillosis [11].

Data on pulmonary aspergillosis in Saudi Arabia is scarce, with almost all reports focusing on allergic-type bronchopulmonary aspergillosis [15]. To the best of our knowledge, no national data on aspergilloma is available. Additionally, our patient was a female in her late thirties who had an occult CPAM, which is generally a rather rare entity to present in adulthood [16] and served as the underlying cause for chronic cavitary pulmonary aspergillosis.

CPAM was first described in 1897 when Stoerk et al. described cystic lesions in a newborn's lung. In 1977, Stoker et al. classified CPAM lesions into types I–III, mainly based on the sizes of the cysts. In 1994, Stocker defined Congenital Pulmonary Airway Malformation (CPAM) as a hamartomatous disease that can arise in any part of the tracheobronchial tree. He further expanded its classification to encompass five types (0–IV) and proposed the term CPAM.

CPAM Type I accounts for nearly 60–65% of cases. The lesion is predominantly cystic, measuring 3–10 cm in diameter, and is surrounded by smaller cysts. Microscopically, the large, thin-walled cysts are lined by ciliated pseudostratified columnar epithelium with some mucin-producing cells. Their walls are composed of fibromuscular and elastic tissue. This type of CPAM is operable and has a good prognosis.

Approximately 80% of CPAMs are detected and diagnosed prenatally or during the neonatal period, when patients initially present with respiratory failure and cyanosis. Most cases of CPAM that are not identified during the neonatal period are detected in infants and school-age children, and these diagnoses are often triggered by infections such as pneumonia. CPAMs are rarely found in adults and usually involve the unilateral lobe of the lung, triggered in the same way as in neonates and children [2,3,17].

This report classified our case as Stoker's Type I CPAM based on its radiographic and pathological findings. The radiographic images show one large cyst with a dimension of 8.6 cm × 7.6 cm. Pathologically, the cyst is ectatic and lined with a pseudostratified ciliated epithelium, and is characterized by the absence of bronchial cartilage and glands. These findings are consistent with the classification of Type I CPAM. To the best of our knowledge, only six cases of pulmonary aspergillosis as a complication of CPAM have been reported [2,[5], [6], [7], [8], [9]]. In our case, radiography confirmed the diagnosis of pulmonary aspergilloma. The characteristic intra cavitary ovoid mass was identified. Macroscopically, the lung lobe contained a cyst with a fungal ball measuring 3 cm by 2.5 cm by 2 cm. Microscopic examination of the fungal ball showed a laminated cut surface and the presence of fungal hyphae by GMS and PAS staining was spotted, mixed with mucous and cellular debris.

Seven cases were reported of patients with CPAM complicated by aspergilloma in the literature, four of which had detailed history of the clinical presentation and the outcome. The predominant presentation of the patients with CCAM complicated with aspergilloma was respiratory symptoms such as cough, sputum production, fever, and dyspnea [2,[5], [6], [7]], in rather healthy adults, with one patient who had asthma and active pulmonary tuberculosis. Our patient, who was healthy in her mid-thirties, also experienced similar symptoms, except for fever. However, the duration of these symptoms in the previously reported cases ranged from 1 week to a few months, whereas our patient had chronic, intermittent symptoms that persisted from early adolescence to her thirties and reported one episode of hemoptysis. Two cases documented hemoptysis in a 14-year-old and 59-year males [5,7], the latter had massive hemoptysis and required bronchial artery angiography embolization followed by lobectomy, however the patient died from hemorrhagic shock secondary to left lobectomy. It is worth noting that This patient had medical history of asthma and 30 years of smoking and was also treated for active pulmonary tuberculosis 7 years prior to his presentation [5].

In two documented cases, antibiotics and antifungal medications were used with little to no effect [5,7]. Additionally, the initial diagnosis of pneumonia based on clinical findings prior to a chest CT scan was not uncommon in the reported cases, which was also the case with our patient. All cases were diagnosed through a chest CT scan, which revealed a cavitary lesion filled with mycetoma. Lobectomy was the preferred management technique after observing no improvement in symptoms or in cases of life-threatening massive hemoptysis in the majority of cases [2,[5], [6], [7], [8], [9]]. One patient, however, experienced improvement with symptomatic treatment, although the specific treatment was not further specified [6]. The cavity type was documented as type 2 in three cases and unreported in the remaining cases [5,6,8].

CT scanning is believed to be a more accurate technique than the standard chest radiography in the recognition of fungus balls, particularly when there is fibrosis and lung distortion [18]. Surgery is the primary choice for treating CPAM and aspergilloma; however, systemic antifungal and steroid treatments have shown limited results [18]. Other therapies include intracavitary instillation of antifungal agents and embolization of bronchial arteries to stop hemorrhage [19].

In conclusion, this case emphasizes the importance of considering radiographic imaging, particularly CT scan, in patients with persistent respiratory symptoms such as asthmatic or allergic-like symptoms unresponsive to treatment, to diagnose unrecognized and potentially complicated malformations of the lung such as CPAM complicated by aspergilloma, even in the absence of neonatal or infancy diagnosis.

## Conflict of interest

There are none.

## Ethical Form

Please note that this journal requires full disclosure of all sources of funding and potential conflicts of interest. The journal also requires a declaration that the author(s) have obtained written and signed consent to publish the case report/case series from the patient(s) or legal guardian(s).

The statements on funding, conflict of interest and consent need to be submitted via our Ethical Form that can be downloaded from the submission site www.ees.elsevier.com/mmcr. **Please note that your manuscript will not be considered for publication until the signed Ethical Form has been received.**
